# Massive Pheochromocytomatosis Treated With Cytoreductive Surgery

**DOI:** 10.1210/jcemcr/luaf306

**Published:** 2025-12-30

**Authors:** Tobias Carling, Alejandra Kalik, Meredith LaRue

**Affiliations:** Carling Adrenal Center, Tampa, FL 33615, USA; Department of Surgery, Hospital for Endocrine Surgery, Tampa, FL 33615, USA; Department of Pathology, Hospital for Endocrine Surgery, Tampa, FL 33615, USA; Carling Adrenal Center, Tampa, FL 33615, USA; Department of Surgery, Hospital for Endocrine Surgery, Tampa, FL 33615, USA

**Keywords:** pheochromocytoma, pheochromocytomatosis, adrenal, adrenalectomy, surgery, cytoreductive

## Abstract

A 35-year-old woman presented in 2022 to the Carling Adrenal Center with recurrent palpitations, anxiety, and diaphoresis, 9 years after right laparoscopic adrenalectomy at a local hospital for pheochromocytoma, diagnosed during pregnancy with placental abruption. Biochemical evaluation revealed markedly elevated 24-hour urinary and plasma fractionated metanephrines. Imaging identified extensive intra-abdominal tumor implants and a solitary liver lesion, raising suspicion for pheochromocytomatosis vs metastatic disease. Genetic testing was negative for major known pathogenic variants. Cytoreductive surgery successfully resected more than 200 tumor deposits, including a 15.5 cm omental mass, without complications. Pathology confirmed pheochromocytomatosis, with the liver lesion identified as focal nodular hyperplasia. Postoperatively, symptoms resolved, biochemical markers significantly improved but remain at ∼2.5- to 3.8 times above normal range, indicating residual microscopic disease (40 months follow-up). This case highlights the feasibility of cytoreductive surgery for extensive pheochromocytomatosis and the challenging distinction between tumor seeding and metastatic pheochromocytoma, emphasizing the need for lifelong biochemical surveillance.

## Introduction

Pheochromocytomas are rare catecholamine-secreting tumors of adrenal chromaffin cells, with an incidence of 0.8 per 100 000 person-years [1, 2]. Recurrence of the disease can occur and is driven by genetic predisposition, loco-regional invasion, lymph node and distant metastases, or iatrogenic tumor seeding, termed pheochromocytomatosis [3, 4]. Recurrence of sporadic pheochromocytoma after curative adrenalectomy is about 3% in intra-adrenal lesions, excluding paragangliomas [5]. In contrast, for sporadic combined pheochromocytoma/paraganglioma cases, the recurrence rate is higher, at 14.7% [4]. Patients with genetic predisposition face even greater risks, with recurrence rates as high as 47.5% in those with pathogenic variants in cluster 1 genes (eg, *SDHx* or *VHL*), compared to 14.9% for cluster 2 gene variants (such as in *RET* or *NF1*). Pheochromocytomatosis results from intraoperative tumor disruption, leading to multifocal tumor cell implantation, distinct from metastatic disease, which involves distant spread to lymph nodes or organs (liver, lung, bone, etc), via lymphovascular invasion or hematogenic dissemination [6, 7]. All pheochromocytomas have metastatic potential, with no definitive histopathological features to predict such behavior. Thus, unequivocal evidence of lymph node or distant organ involvement is required for the diagnosis of metastatic pheochromocytoma [8, 9]. Differentiating between pheochromocytomatosis and metastatic pheochromocytoma is critical but challenging, often requiring comprehensive biochemical, imaging, and histopathological evaluation [10, 11]. The importance of the distinction relates to the treatment (frequently nonsurgical in metastatic pheochromocytoma) and prognosis, as metastatic disease has worse outcomes. We describe a patient with massive pheochromocytomatosis treated with cytoreductive surgery, highlighting the feasibility of extensive resection and the importance of distinguishing iatrogenic tumor seeding from metastatic pheochromocytoma. It emphasizes meticulous surgical technique to prevent pheochromocytoma tumor implantation and lifelong surveillance to detect recurrence, contributing to the sparse literature on this rare condition [9, 12].

## Case Presentation

A 35-year-old woman presented to the Carling Adrenal Center (CAC) in January 2022 with adrenergic symptoms including palpitations, irritability, anxiety, pallor, tremor, and diaphoresis. Nine years earlier, in 2013 at age 27, she underwent right laparoscopic transabdominal adrenalectomy (LTA) elsewhere for pheochromocytoma diagnosed during pregnancy complicated by cardiomyopathy, labile hypertension, placental abruption, and successful cesarean delivery. Operative and pathology reports showed intraoperative bleeding requiring hemoclips, and gross fragmentation of the 4.2 × 3.8 cm tumor without capsular/vascular invasion or necrosis; mitotic figures were rare, with positive synaptophysin, chromogranin, and S-100 immunohistochemistry. Symptoms resolved for 2 years after surgery. In 2015, palpitations led to a negative cardiac workup without further evaluation. No pheochromocytoma surveillance (genetic, biochemical, or imaging) occurred from 2013 to 2021. In late summer 2021, recurrent spells (3-4 times daily, mainly nocturnal, lasting 5-10 minutes) prompted consultations with a local endocrinologist and oncologist. Her medical, pharmaceutical, surgical, and family history were otherwise non-contributory. On physical examination, her blood pressure was 126/86 (she never had a baseline elevated blood pressure), pulse 90, 163 cm, 48.5 kg (body mass index [BMI] of 18.4 kg/m^2^), with well-healed incisions from her right LTA.

## Diagnostic Assessment

**Figure 1.**
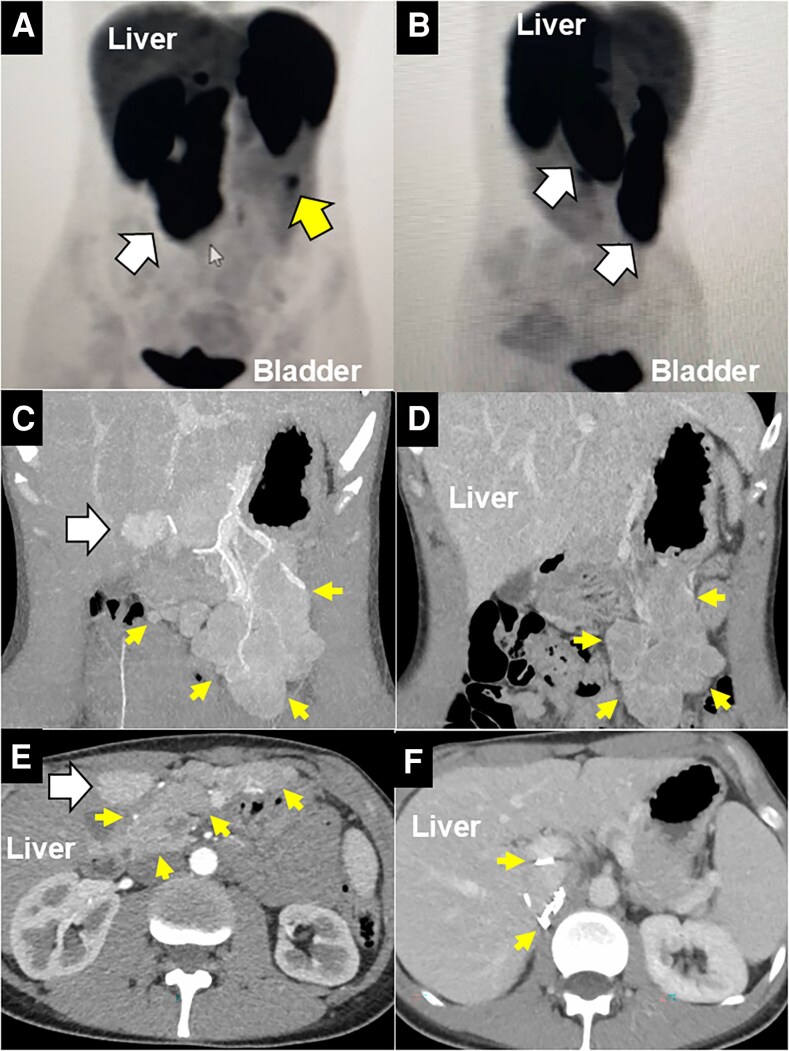
Preoperative imaging demonstrating massive pheochromocytomatosis. A-B, ^68^Ga-DOTATATE PET scan performed at the patient's home institution, displaying large areas of intra-abdominal uptake (white arrow, A coronal, B sagittal views) in the right upper quadrant, as well as smaller areas on the left side (yellow arrow). Physiological uptake is noted in the liver and urinary bladder. C-D, Coronal CT images of arterial (C) and venous phase (D) demonstrating numerous multi-loculated, highly vascular intra-abdominal lesions (mainly in the omentum and in the right adrenalectomy bed; yellow arrows), and one solitary liver lesion in segment IV (white arrow). E-F, Axial CT images of arterial (E) and venous phase (F) demonstrating numerous multi-loculated, highly vascular intra-abdominal lesions (yellow arrows), and one solitary liver lesion in segment IV (E, white arrow), and, more cephalad, evidence of numerous clips (F) and tumor deposits in the right adrenalectomy bed (yellow arrows). All CT scan images depicted are according to a modified adrenal protocol of the Carling Adrenal Center and Hospital for Endocrine Surgery.

The biochemical assessment was initiated locally in the fall of 2021 and revealed 24-hour urine fractionated metanephrines 4149 μg/24 h (SI: 21.1 μmol/24 h; reference range: 36-229 μg/24 h; SI: <1.6 μmol/24 h), normetanephrines 3695 μg/24 h (SI: 20.2 μmol/24 h; reference range: 95-650 μg/24 h; SI: <2.1 μmol/24 h; via high-performance liquid–chromatography-mass spectrometry [HPLC-MS]; ARUP Laboratories), and plasma metanephrines and normetanephrines 5.70 nmol/L (reference range: 0.00-0.49 nmol/L) and 18.65 nmol/L (reference range: 0.00-0.89 nmol/L), respectively. The 24-hour urine levels were repeated about 6 weeks later and were unchanged. All other routine laboratory studies were normal. Imaging with ^68^gallium (^68^Ga)-DOTATATE positron emission and computed tomography (PET/CT) scan (Fig. 1A and 1B) revealed postsurgical changes with multiple surgical clips in the right adrenalectomy bed with uptake consistent with residual tumor tissue. For remedial surgery planning, these clips may signal extensive scarring and potential tumor implantation near the inferior vena cava (IVC), making the dissection treacherous due to dense adhesions, and heightened vascular injury risk. There was increased activity posterior to the site of previous surgery in the aortocaval region, as well as massive uptake in the anterior mesentery, with possible residual mass, or bulky lymphadenopathy. There was also a solitary lesion in segment IV of the liver that was concerning for metastatic disease. To obtain exquisitely detailed anatomical depiction of the tumor implants, a modified (focused on the vasculature, with reduced phases to limit ionizing radiation exposure) adrenal protocol CT scan was performed (Fig. 1C-1F). Genetic testing (Ambry Genetics, 77-gene panel) was negative for pathogenic variants in known pheochromocytoma/paraganglioma genes, including TMEM127, but cannot detect rare chromosomal alterations, duplications, or fusions seen in some pheochromocytomatosis cases [6, 13]. Preoperative diagnosis favored pheochromocytomatosis from tumor seeding during initial right LTA, although the solitary segment IV liver lesion raised metastatic concern. This was supported by: (1) imaging showing isolated liver lesion without lung/bone metastases, suggesting localized process; (2) likely seeding mechanism from surgical clips and disrupted tumor on pathology; (3) peritoneal-dominant implant distribution, consistent with iatrogenic spread; and (4) absent pathogenic germline variants, favoring sporadic etiology. However, these factors are supportive but not conclusive for distinguishing pheochromocytomatosis from metastatic disease. Furthermore, it may be difficult to distinguish between pheochromocytomatosis and metastatic pheochromocytoma in some cases, and there may be instances of overlapping pathologies, such as a combination of the 2 conditions.

## Treatment

**Figure 2.**
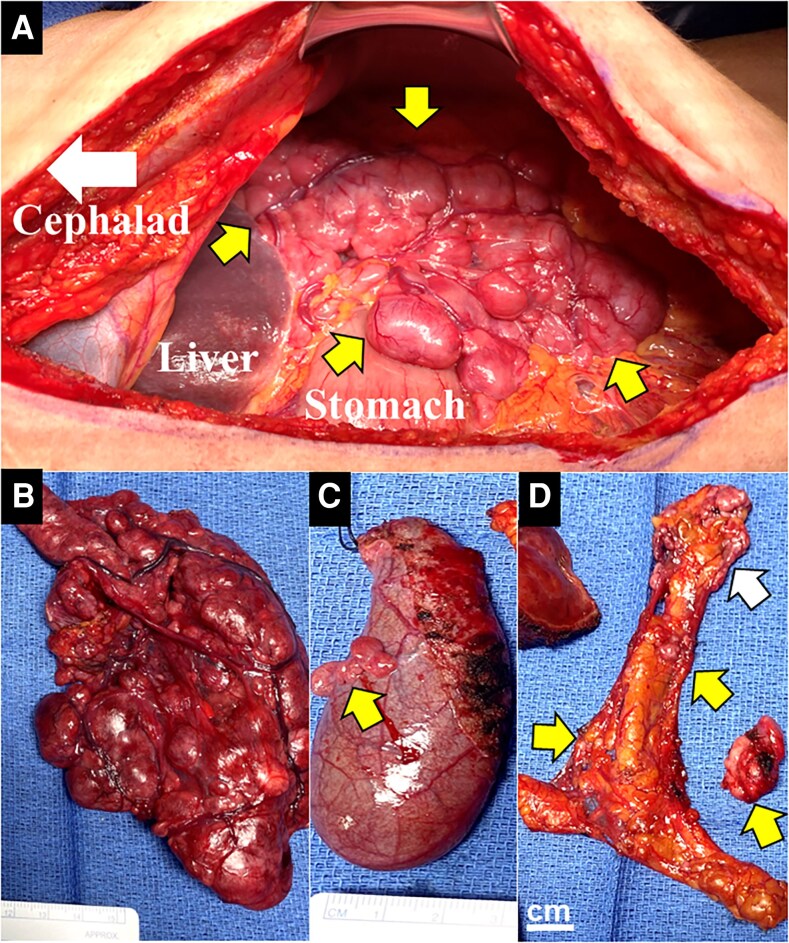
Intraoperative findings during cytoreductive surgery for massive pheochromocytomatosis. A, The maximally invasive midline laparotomy incision provided appropriate access to numerous multi-loculated, highly vascular intra-abdominal lesions (mainly in the omentum and in the right adrenalectomy bed; yellow arrows). B, An en bloc resection of the largest contiguous mass (15.5 × 9.0 × 4.0 cm from the omentum. C-D, Numerous tumor deposits were identified on the gallbladder surface (C, yellow arrow), as well as in additional areas of omental tissue (D, yellow arrows), along with tumor deposits in the right adrenalectomy bed (D, white arrow), which were all resected in a cytoreductive manner. Additional tumor deposits were identified and resected in the peri-pancreatic space, lesser sac, and on the liver surface (not shown).

The patient tolerated doxazosin 2 mg twice a day for 2 weeks prior to surgery. Using a midline laparotomy incision, the entire abdominal cavity was evaluated (Fig. 2), with the goal of the cytoreductive operation to resect all gross disease. The intraoperative findings were consistent with pheochromocytomatosis with numerous implants (>200 grossly visible lesions, ranging in size from 0.1-1.5 cm) throughout the abdominal cavity. The largest 15.5 × 9.0 × 4.0 cm omental mass was resected en bloc; deposits, primarily in omentum, right adrenalectomy bed, peri-pancreatic/splenic spaces, lesser sac, aortocaval area, and liver/gallbladder surfaces, were fully removed, including omentectomy and cholecystectomy. The implants were soft, nonadherent, and easily dissected, unlike typically adherent metastatic pheochromocytomas due to a desmoplastic reaction [12]. The solitary segment IV liver lesion was removed via wedge resection. No significant hemodynamic fluctuations, blood loss, vasopressors, or ICU admission occurred. The patient recovered well and was discharged on postoperative day 2 on oral pain medications.

## Outcome and Follow-Up

**Figure 3:**
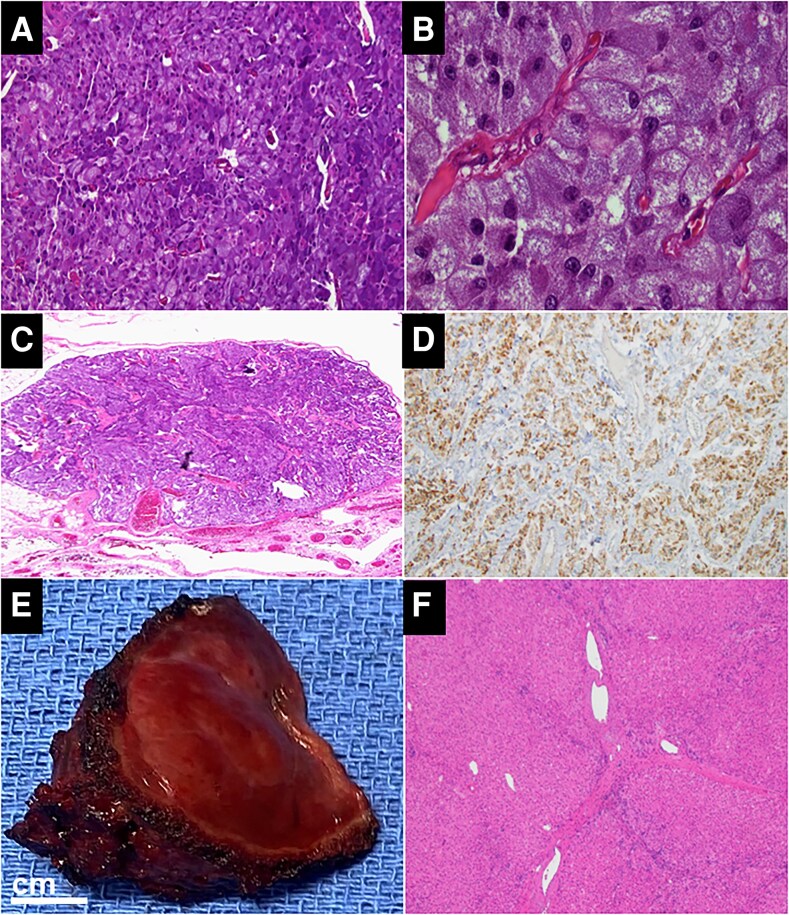
Gross and histopathology from a case with massive pheochromocytomatosis. Representative images from surgical pathology showing typical pheochromocytoma tumor cells from the largest 15.5 × 9.0 × 4.0 cm omental mass (A and B), as well as a representative (out of >200 grossly visible lesions) smaller right adrenal bed tumor deposits (C). Staining used hematoxylin and eosin ([HE] A-C) as well as SDH immunostaining (D, showing retained expression), using magnification of 40x (A and C), 100x (B and D). Further immunostaining revealed positivity for synaptophysin, Chromogranin, S100, and a low KI-67-proliferative index of 2% (not shown). E-F, Gross pathology of the liver wedge resection of the segment IV liver lesion (4.0 × 3.5 × 2.0 cm), and HE staining using magnification of 40x, demonstrating typical histology of focal nodular hyperplasia of the liver without any evidence of pheochromocytoma tumor cells.

Pathology confirmed pheochromocytomatosis (Fig. 3), with the 15.5 × 9.0 × 4.0 cm omental mass and numerous smaller deposits (0.1-1.5 cm) showing insipid pheochromocytoma cells with retained synaptophysin, chromogranin, S-100, *SDHB* expression, and 2% Ki-67 index. All intraoperative lesions (omentum, right adrenalectomy bed, peri-pancreatic/splenic spaces, lesser sac, aortocaval area, liver/gallbladder surfaces) were pheochromocytoma deposits. Importantly, all lymph nodes were negative, and the segment IV liver lesion was focal nodular hyperplasia, and unrelated to the pheochromocytomatosis (Fig. 3E and 3F). Moreover, all the tumor implants of the liver were located on the surface and not within the hepatic parenchyma. Collectively, findings supported iatrogenic seeding over metastatic pheochromocytoma. Postoperatively, symptoms resolved, and at 40 months, the patient remains asymptomatic. The plasma metanephrines significantly improved (>90% drop) but have remained at ∼2.5- to 3.8-fold above normal upper limit of the reference range, suggesting residual microscopic disease. A follow-up ^68^Ga-DOTATATE PET/CT scan, performed 14 months after cytoreductive surgery, was negative except for nonfocal uptake in the area of the right adrenalectomy bed from the 2013 operation (Fig. 1F). This outcome was exceedingly favorable given the extensive disease burden, highlighting the efficacy of cytoreductive surgery [9, 14].

## Discussion

Pheochromocytomatosis, a rare cause of pheochromocytoma recurrence, results from iatrogenic tumor seeding during initial surgery, and likely occurred by tumor fragmentation in this patient's prior LTA [4, 7]. Such cases highlight the importance of meticulous surgical techniques, including gentle handling and avoiding tumor rupture to prevent spillage during pheochromocytoma resections, regardless of the surgical approach used (eg, the mini back scope adrenalectomy, posterior retroperitoneoscopic, LTA, or open techniques) [12]. The presence of numerous surgical clips, compared to the typical 1 to 2 used to ligate the right adrenal vein in an uncomplicated operation, suggests a difficult initial LTA in which the tumor capsule may have been penetrated, releasing viable cells into the surgical field, causing pheochromocytomatosis. Unlike metastatic pheochromocytoma, which involves distant spread to lymph nodes or organs, pheochromocytomatosis is characterized by multifocal implants, typically in the peritoneal cavity [6, 13]. The critical distinction between these entities is challenging, as both may present with similar biochemical and imaging findings, but histopathology, showing no lymph node involvement or features indicative of lymphatic or vessel dissemination to distant organs, as well as intraoperative findings, confirms pheochromocytomatosis, as in this case [9, 11]. The absence of genetic mutations in known tumor susceptibility genes further supports the mechanism of iatrogenic tumor seeding as the cause of recurrence, contrasting with typically *SDHB*-related metastatic pheochromocytoma, although not all metastatic cases have mutations in *SDHB*, or other known driver genes [10, 11].

The diagnosis of pheochromocytomatosis requires a high index of suspicion, as symptoms like palpitations and diaphoresis mimic primary pheochromocytoma and may be delayed by years [4, 15]. Advanced imaging, such as ^68^Ga-DOTATATE PET/CT, is useful for mapping implants, although the initial concern for liver metastasis underscores diagnostic complexity in distinguishing the mechanism of recurrent disease [11, 16, 17]. The confirmation of focal nodular hyperplasia as opposed to metastatic pheochromocytoma tumor deposits in the liver highlights the necessity of surgical exploration in this case to achieve a definite clinical and pathological diagnosis [8]. The presumption of metastatic pheochromocytoma based on imaging alone had led to the clinical recommendation to forego any surgical attempts in lieu of systemic chemotherapy at another facility [18, 19].

Cytoreductive surgery is the mainstay of treatment of pheochromocytomatosis, with this case demonstrating its feasibility in resecting more than 200 implants without complications [12, 20]. Unlike metastatic pheochromocytomas, which are often adherent due to a desmoplastic reaction and surgically challenging, these implants were easily dissected, facilitating complete gross en bloc resection [21, 22]. Persistent biochemical abnormalities suggest residual microscopic disease, a common outcome due to the inability to eliminate all tumor cells [1, 14]. Adjunctive therapies may be considered as she is likely to develop recurrent, symptomatic disease, although this may take several years [9, 14, 16, 23]. Although there is a lack of data on long-term outcomes due to the rarity of the disease, pheochromocytomatosis seems to have a favorable prognosis with resection, whereas metastatic pheochromocytoma has a 5-year survival of 50% to 80% depending on rate of progression and metastatic site [9, 11, 18, 19]. The limitations of the study include the lack of surveillance after the initial surgery, delaying diagnosis, and incomplete details of the 2013 procedure, limiting insights into seeding mechanisms [4, 15]. Lifelong annual biochemical monitoring is critical to detect recurrence early, mitigating risks of catecholamine excess [9]. This case underscores the need for meticulous surgical technique during pheochromocytoma surgery, regardless of surgical approach, to prevent seeding [12, 22]. In the modern era of mini back scope adrenalectomy, pheochromocytomas can frequently be removed without tumor spillage in less than 20 minutes, with minimal complications, and without need for intensive care management [12].

## Learning Points

- Pheochromocytomatosis is a rare recurrence mechanism due to intraoperative tumor seeding, distinct from metastatic pheochromocytoma.
- Symptoms mimic primary pheochromocytoma, necessitating a high index of suspicion and thorough endocrine evaluation.
- Cytoreductive surgery is feasible and effective for symptom control, although residual microscopic disease may persist.
- Lifelong annual biochemical surveillance is crucial to detect recurrence early and prevent complications.

## Contributors

All authors made individual contributions to authorship. T.C., A.K., and M.L. were responsible for data collection, analysis, diagnosis, and management of the patient. T.C. performed the cytoreductive surgery, obtained intraoperative and gross pathology images, and wrote the initial draft, and A.K. prepared the histology images. All authors were involved in manuscript preparation reviewed and approved the final draft.

## Data Availability

Data sharing is not applicable to this article as no datasets were generated or analyzed during the current study.

## Abbreviations

CT: computed tomography
LTA: laparoscopic transabdominal adrenalectomy
PET: positron emission tomography
